# 

**Published:** 1801-09

**Authors:** 


### LECTURES.

### *Medical School of St. Thomas's and Guy's Hospitals.*

The Winter Course of Lectures at these united Hospitals will commence in the following order.

Anatomy and Surgery, by Mr. CLINE and Mr. A. COOPER, on Thursday, October 1, at one o'clock.

Practice of Medicine, by Dr. BABINGTON, on Friday, Oct. 2, at ten o'clock in the morning.

Midwifery and Diseases of Women and Children, by Dr. LOWDER and Dr. HAIGHTON, on Saturday, Oct. 3, at eight in the morning.

Chemistry and Experimental Philosophy, by Dr. BABINGTON and the Rev. Mr. ROBERTS, on the same morning, at ten.

Physiology, or Laws of the Animal Œconomy, by Dr. HAIGHTON, on Monday, Oct. 5, at a quarter before seven in the evening.

Theory of Medicine and Materia Medica, by Dr. CURRY, on Tuesday, Oct. 6, at seven in the evening.

Principles and Practice of Surgery, (illustrated by select cases under his care in the Hospital) by Mr. ASTLEY COOPER, on Monday, Oct. 12, at eight in the evening.

In addition to these, Dr. SAUNDERS will, early in October, begin a Course of Clinical Lectures on select Medical Cases under his care in the Hospital.

The Lectures given at these Hospitals are so arranged, that no two of them interfere with each other in the hours of attendance; and the whole is calculated to form a complete circle of Medical Instruction.

N. B. Mr. FOX, in the Course of the Season, will deliver his Lectures upon the Structure and Diseases of the Teeth.

Terms and other particulars to be had at the Hospitals, or of Mr. Cox, bookseller, St. Thomas's Street, Southwark.

---

### *Medical Theatre, St. Bartholomew's Hospital.*

The following Courses of Lectures will be delivered at this Hospital during the ensuing season.

On the Theory and Practice of Medicine, by Dr. ROBERTS.

On

On Anatomy and Physiology, by Mr. ABERNETHY.
On Comparative Anatomy, by Mr. MACARTNEY.
On the Theory and Practice of Surgery, by Mr. ABERNETHY.
On Chemistry and the Materia Medica, by Dr. POWELL.
On Midwifery and the Diseases of Women and Children, by Dr. THYNNE.

---

*Medical Theatre, London Hospital.*

The Lectures on Anatomy, Physiology, and the Principles and Operations of Surgery, with practical Anatomy, as usual, will commence on the 1st of October, by Mr. BLIZARD, Mr. J. BLIZARD, and Mr. HEADINGTON, Surgeons of the Hospital.

---

The Medical Lectures in the *University of Glasgow* will begin on Tuesday the 3d of November, at the following hours.

Dietetics, Materia Medica, and Pharmacy, by Dr. MILLAR, at ten o'clock in the forenoon.

Midwifery, by Mr. TOWERS, at eleven o'clock.

Theory and Practice of Physic, by Dr. FREER, at twelve.

Anatomy and Surgery, by Dr. JEFFRAY, at two o'clock in the afternoon.

Chemistry and Chemical Pharmacy, by Dr. CLEGHORN, at seven o'clock.

Clinical Lectures on the cases of patients in the Royal Infirmary, by Dr. FREER and Dr. CLEGHORN. The first Lecture by Dr. Freer, on Thursday, the 12th of November, at six o'clock in the evening.

Dr. BROWN will begin his Lectures on Botany about the beginning of May next.

---

*Lectures at the St. Mary-le-Bone Infirmary.*

On the Principles of Medical Science, by Dr. ROWLEY.
On the Practice of Physic and Morbid Anatomy, by Dr. HOOPER.

---

Dr. CRICHTON will commence his Autumnal Course of Lectures on Medicine and Chemistry, at his Lecture Room in Clifford-street, Bond-street, on Monday, October 12. The Lectures on the Theory and Practice of Physic will be delivered every morning, Sundays excepted, at eight o'clock; those on Chemistry every Monday, Wednesday, and Friday, at nine; and those on Materia Medica every Tuesday, Thursday, and Saturday, at the same hour. Further particulars may be obtained by applying to Dr. C. at his house, No. 15, Clifford-street.

---

Dr. BRADLEY will commence his Autumnal Course of Lectures on the Theory and Practice of Medicine, on Monday, Oct. 5, at the Lecture Room, No. 102, Leadenhall-street, at six in the afternoon. The Course will consist of about Seventy Lectures. Terms,

Three

Three Guineas. Private Courses, for the accommodation of young Surgeons in the Navy or Army, are continued every morning at Dr. Bradley's, Delahay-street, Westminster.

A Course of Lectures will be delivered on one or two evenings in the week, during the season, on the Principles of Chemistry and their application to Pharmaceutical Processes, by a Practical Chemist.

---

Dr. Garnet intends to deliver Lectures on the Theory and Practice of Medicine, and on the Animal Œconomy; he also proposes to deliver Lectures on Chemistry, and those branches of Natural Philosophy particularly connected with Medicine. These different Courses will commence in October next, at his house, No. 51, Great Marlborough Street.

---

Dr. Osborn's and Dr. Clarke's Lectures upon the Principles and Practice of Midwifery, and the Diseases of Women and Children, will begin on Friday, October 2. They will be given in future only at Dr. Clarke's house in New Burlington-street, from half past ten to half past eleven in the morning, for the convenience of gentlemen attending the different Hospitals.

---

Dr. Batty's Lectures on Midwifery, and on the Diseases of Women and Children, will commence at his house in Marlborough Street, on Monday, October 5, at eleven o'clock in the morning.

---

Dr. Dennison and Dr. Squire, Physicians and Men-Midwives to the Lying-In Charity for delivering poor Women at their own habitations, will begin a Course of Lectures on Thursday, October the 1st, on the Theory and Practice of Midwifery and the Diseases of Women and Children.—In addition to a constant supply of labours for practical improvement, gentlemen will have frequent opportunities of visiting patients in disorders during pregnancy, and the advantage of seeing the treatment of diseases incident to the state of child-birth and early infancy.

---

Mr. Pearson, Surgeon of the Lock Hospital and Asylum, and of the Public Dispensary, will begin his Autumnal Course of Lectures on the Principles and Practice of Surgery, on Monday, October 5, at seven o'clock in the evening.—In this Course he will deliver an extensive account of the history and treatment of Scrofula and Lues Venerea.

---

On the 5th day of October, Mr. T. Pole will commence his Course of Lectures on the Theory and Practice of Midwifery, including the Diseases of Women and Children, at his house, No. 102, Leadenhall-street, near the Royal Exchange, where printed particulars may be had.

---

Mr,

Mr. CHEVALIER, Surgeon to the Westminster General Dispensary, will commence his Autumnal Course of Lectures on the Principles and Operations of Surgery, on Monday the 5th of October, at seven o'clock in the evening, at his house, No. 20, South Audley-street, Grosvenor-square, where a printed Plan of the Course may be had, or at the Dispensary in Gerard-street, Soho.

---

Mr. H. LEIGH THOMAS will commence his Winter Course of Lectures on the Principles and Practice of Surgery, on Monday, October 5; in which the different operations will be performed, and the medical treatment fully explained.—Further particulars may be known at his house in Leicester-square, or at the Anatomical Theatre, Windmill-street.

---

At the Theatre of Anatomy, Great Windmill-street, Two Courses of Lectures are read by Mr. WILSON during the Winter and Spring Seasons; one Course beginning on the 1st Day of October, and terminating on the 18th day of January; the other Course beginning on the 19th day of January, and terminating towards the middle of May.

In each Course is explained the structure of every part of the human Body, so as to exhibit a complete view of its Anatomy, as far as it has been hitherto investigated; to which are added, its Physiology and Pathology; after which follow Lectures on the Operations of Surgery; and the Course concludes with the Anatomy of the Gravid Uterus.

A Lecture is given daily from two until four o'clock in the afternoon.

A room is likewise open for Dissections, under the direction of Mr. WILSON, and Mr. THOMAS, from nine o'clock in the morning till two in the afternoon, from the 10th day of October till the 20th of April; where regular and full demonstrations of the Parts are given; where the different cases in Surgery are explained, the methods of Operating shewn on the dead Body; and where also the various arts of injecting and making Preparations are taught.

Further particulars may be known by applying to Mr. Wilson, at the Anatomical Theatre; or at his house in Argyll-street, Hanover Square; or to Mr. Thomas, Leicester Square.

---

Mr. CARPUE will commence his Anatomical Lectures, at his Theatre, Broad Street, Golden Square, on Monday, the 5th of October, 1801. The Structure of the human Body will be explained, as also its Physiology. The Operations of Surgery will be shown on the dead body. Mr. C's plan prevents him from taking more than ten Pupils. Every day some part of the human body is demonstrated to the pupils, who are required to demonstrate it afterwards to him. Myology is not taught till the pupils are perfectly instructed in Osteology, &c. &c. A general examination takes place every tenth day; and if the pupils do not perfectly recollect recollect any of the parts that have been lectured on, they are again demonstrated. The Dissecting Room will be open from nine o'clock in the morning till half past two. The pupils are here taught the method of operating, as also the art of making preparations.

Further particulars may be known by applying to Mr. Carpue, at his house, No. 44, Leicester Square.

---

ERRATUM.

*In Dr. Beddoes's Letter, in our last Number, p.* 165, *before* occasioned, *insert* has not.

---

### To the PURCHASERS of the MEDICAL and PHYSICAL JOURNAL.

*NOTWITHSTANDING every periodical Publication has been raised in its price since the late* DOUBLE DUTY *upon paper, and the advance of* 30 *per cent. in the expence of printing, the Medical and Physical Journal has hitherto been continued at its original price. The Proprietor had a stock of paper in his possession at the old duty, and he was unwilling to advance this work till compelled to purchase a fresh supply of paper at the new prices.*

*The advance in the paper upon which this work is printed, proves to be* FIFTEEN SHILLINGS *per ream, creating an additional expence to the Proprietor, in this single article, of upwards of twenty pounds per month; he is compelled therefore by necessity to add* SIX PENCE *to the price of the Work, and in future to sell all the Numbers at* HALF A CROWN. *Nothing has given him more pain than to be obliged thus to advance the price of the Work; he hopes, however, that the reasonableness of this measure will be apparent, and that no professional man will deprive himself of the valuable information disseminated in* THE MEDICAL AND PHYSICAL JOURNAL, *by the present unavoidable advance in its price.*

---

*Persons who reside abroad, and who wish to be supplied with this Work every month as published, may have it sent to them,* FREE OF POSTAGE, *to New York, Halifax, Quebec, and every part of the West Indies, at three guineas per annum, by Mr.* THORNHILL, *of the General Post Office, at No.* 21, *Sherborne Lane; to Hamburgh, Lisbon, Gibraltar, and every part of the Mediterranean, at three guineas per annum, by Mr.* BISHOP, *of the General Post Office, at No.* 22, *Sherborne Lane; to the Cape of Good Hope, or to any part of the East Indies, at two guineas per annum, by Mr.* GUY, *of the India House; and to any part of Ireland, at two pounds ten shillings per annum, by Mr.* SMITH, *of the General Post Office, at No.* 3, *in Sherborne Lane. It may also be had of all persons who deal in Books in every part of the world.*

